# Taccalonolides: A Novel Class of Microtubule-Stabilizing Anticancer Agents

**DOI:** 10.3390/cancers13040920

**Published:** 2021-02-22

**Authors:** Xiaoyan Chen, Angela Winstead, Hongtao Yu, Jiangnan Peng

**Affiliations:** Department of Chemistry, Morgan State University, Baltimore, MD 21251, USA; xiaoyan.chen@morgan.edu (X.C.); Angela.Winstead@morgan.edu (A.W.); hongtao.yu@morgan.edu (H.Y.)

**Keywords:** taccalonolide, microtubule stabilizer, anticancer agent, drug resistance

## Abstract

**Simple Summary:**

Natural products have continued to play an important role in new drug discovery with a considerable number of marketed drugs being derived from naturally occurring compounds, particularly in the area of cancer. Taccalonolides are a new class of microtube-stabilizing agents isolated from plants of the genus *Tacca* demonstrating effectiveness against drug-resistant tumors in cellular and animal models. This review article highlights the discovery history of taccalonolides and their microtubule-stabilizing activities, which summarizes the naturally derived and semi-synthesized structures that have been reported so far and the advances on the mechanism of action of taccalonolides.

**Abstract:**

Microtubule stabilizing agents, such as paclitaxel, docetaxel, and cabazitaxel have been among the most used chemotherapeutic agents in the last decades for the treatment of a wide range of cancers in the clinic. One of the concerns that limit their use in clinical practice is their intrinsic and acquired drug resistance, which is common to most anti-cancer chemotherapeutics. Taccalonolides are a new class of microtubule stabilizers isolated from the roots of a few species in the genus of *Tacca*. In early studies, taccalonolides demonstrated different effects on interphase and mitotic microtubules from those of paclitaxel and laulimalide suggesting a unique mechanism of action. This prompts the exploration of new taccalonolides with various functionalities through the identification of minor constituents of natural origin and semi-synthesis. The experiments on the new highly potent taccalonolides indicated that taccalonolides possessed a unique mechanism of covalently binding to the microtubule. An X-ray diffraction analysis of a crystal of taccalonolides AJ binding to tubulin indicated that the covalent binding site is at β-tubulin D226. Taccalonolides circumvent all three mechanisms of taxane drug resistance both in vitro and in vivo. To improve the activity, the structure modification through semi-synthesis was conducted and the structure-activity relationships (SARs) was analyzed based on natural and semi-synthetical taccalonolides. The C22–C23 epoxide can significantly increase the antiproliferation potency of taccalonolides due to the covalent link of C22 and the carboxylic group of D226. Great progress has been seen in the last few years in the understanding of the mechanism of this class of microtube-stabilizing agents. This review summarizes the structure diversity, structure-activity relationships (SARs), mechanism of action, and in vivo activities of taccalonolides.

## 1. Introduction

**Figure 1:**
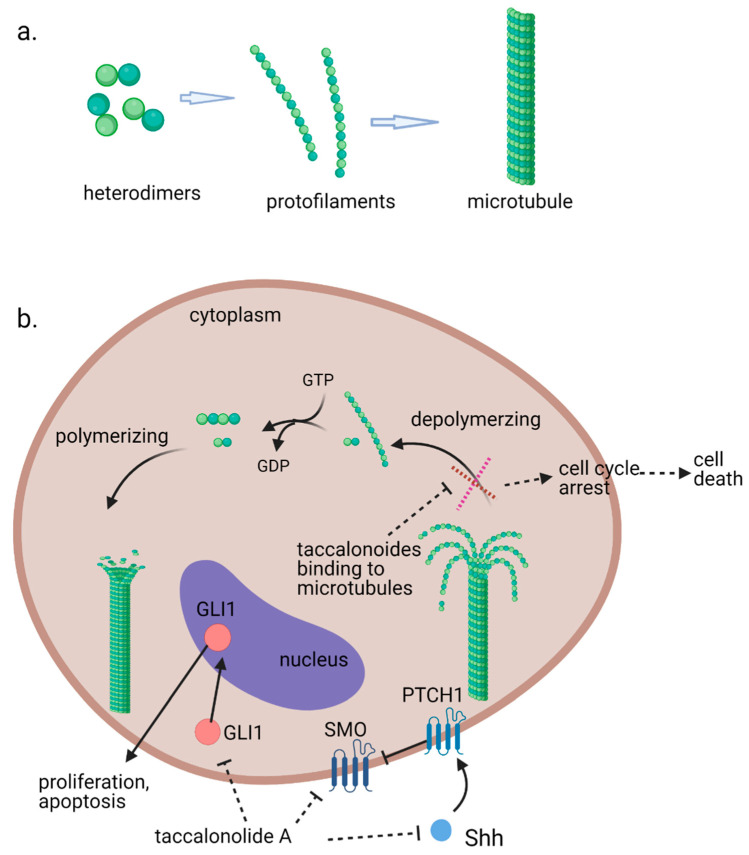
(**a**). Fundamental aspects of microtubule structure. (**b**). The dynamic behavior of microtubules and mechanism of action of microtubule stabilizing agents e.g., taccalonolides. In a recent study, it is observed that mRNA and protein expression levels of sonic hedgehog (Shh), SMO and GlI1 were decreased in HepG2 and Huh7 cells treated with taccalonolide A. polarity. Aberrant activation of the Shh pathway has been shown in a variety of human cancers. Blockage of Shh pathway induces inhibition of downstream protein smoothened (SMO) at the primary cilium resulting in the decreased nuclear localization of glioma-associated (GLI) transcription factors, preventing the activation of GLI target genes [14]. Diagrams were created with BioRender.com (accessed on 12 February 2021).

Microtubules, the third principal component of the cytoskeleton, are tube-like long hollow cylindrical filaments found in all eukaryotes and function in a variety of cell movements including transport of organelles, vesicles, or signaling molecules and the separation of chromosomes during mitosis [1,2]. It has been one of the most successful targets for anticancer drugs including taxanes and vinca alkaloids [3,4]. Microtubules are composed of a single type of globular protein, called tubulin. The heterodimers of α- and β-tubulin subunits string together to make linear strands named protofilaments in a specific head-to-tail orientation that gives microtubules an innate polarity (Figure 1a) [5,6,7]. In mammalian cells, microtubules generally consist of 13 linear protofilaments arranged in parallel. A characteristic property of microtubules is their ability to undergo continual and rapid cycles of assembly (growth) and disassembly (shrinkage) by adding and subtracting tubulin dimers at both ends of the filament, which is known as dynamic instability [5]. The guanine nucleotide molecule (i.e., GTP) bound to the β-tubulin molecule plays a key role in the dynamic instability. The GTP is hydrolyzed to GDP during or shortly after polymerization. This GTP hydrolysis weakens the binding affinity of tubulin to adjacent molecules and renders the microtubules prone to depolymerization [2,8]. During mitosis, the microtubule array present in interphase cells disassembles and the free tubulin subunits are reassembled to form the mitotic spindle, which is responsible for the separation of daughter chromosomes [2]. Because of the central role of the rapid reorganization of microtubules in mitosis, microtubules serve as an important and effective target for anticancer drugs (Figure 1b) [9].

The microtubule-targeted agents, also referred to as microtubule-binding agents, microtubule-active agents, or antimitotic agents, are often classified into two main groups, the destabilizers and the stabilizers, according to their effects on microtubule polymer mass at high concentrations. The microtubule-stabilizing agents enhance microtubule polymerization at high concentrations and most bind to the same or an overlapping taxane-binding site on β-tubulin such as paclitaxel and docetaxel. Another unique binding site, the peloruside/laulimalide-binding site on α-tubulin was also reported [9,10]. Both microtubule destabilizing and stabilizing agents can suppress microtubule dynamicity, which leads to apoptotic cell death [3,11]. 

In an effort to clarify the bitter principles of a medicinal plant *Tacca plantaginea*, the structures of isolated taccalonolide A and taccalonolide B were identified in 1987 [12]. The microtubule-disturbing effects of taccalonolides were first discovered in a study conducted by Dr. Mooberry in 2003 following a positive screening result [13]. The taccalonolides have been extensively investigated since then because of their microtubule-stabilizing properties and their effectiveness against drug-resistant cancers in both in vitro and in vivo models. This review covers the structures of the naturally derived and semi-synthetic taccalonolides reported as of January 2021 and their therapeutic properties that are associated with anticancer actions. An exhaustive literature search for this review was conducted by searching the key words of “taccalonolide”, “microtubule stabilizing”, “microtubule stabilizer”, and “microtubule-binding” on Google Scholar, PubMed and Scifinder. Dr. Mooberry has published a series of reports and patents on taccalonolides in the last ten years. Searches based on the authors from Dr. Mooberry’s group were also performed. The collected literature was organized and analyzed according to the year of publication and study topics such as chemical structures, structural modification, therapeutic properties and mechanism of action of taccalonolides. When it came to choosing an example to articulate a conclusion, the classic studies that played an important role in understanding taccalonolides were given priority in the way that shows the readers an overall clear path of the discovery of taccalonolides. For example, taccalonolides AF and AJ were selected, rather than other more potent molecules, to demonstrate the significance of C22–23 epoxy group in structure-activity relationships given that their discovery was among the leap forward in the research of taccalonolides and they were used extensively in subsequently studies.

## 2. Structures of Natural Taccalonolides (1987–2020)

**Figure 2:**
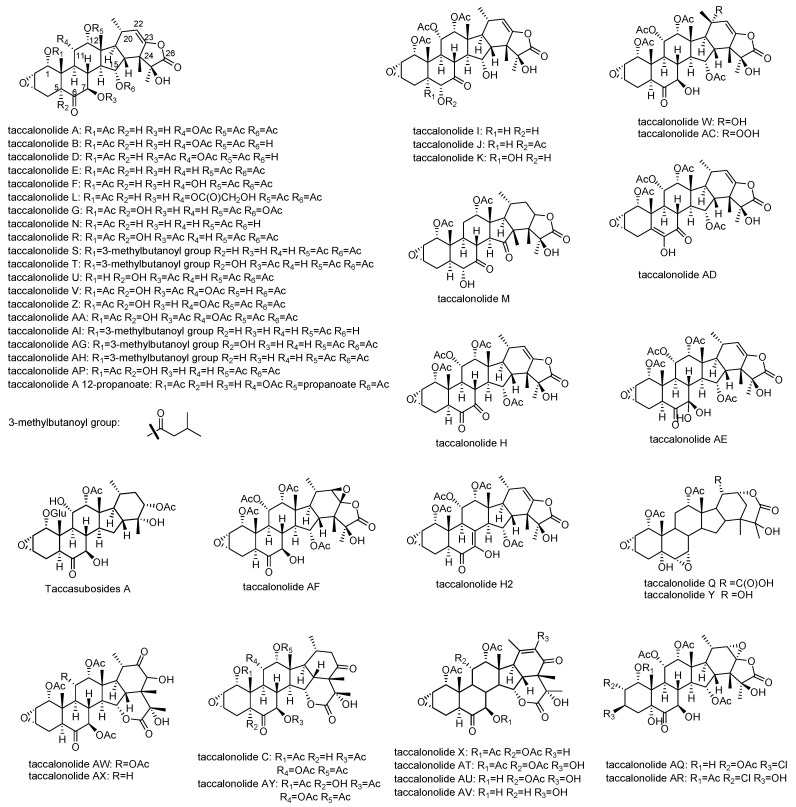
Structures of naturally derived taccalonolides.

Taccalonolides are a group of highly acetoxylated pentacyclic steroids containing 28 carbons isolated from plants of the genus *Tacca* (Table 1). The typical taccalonolides have a C2–C3 epoxide group and an enol-γ-lactone fused with the unique E ring (Figure 2) [15]. The majority of the structural variations of taccalonolides are due to oxidation or acetoxylation at the backbone of 6-6-6-5-6-fused rings. The most numerous taccalonolides are those containing either an acetoxy or a hydroxy group at positions C1, C7, C11, C12, and C15. Most of these compounds also contain a hydroxy group at C5 such as taccalonolides G and U. A small number of taccalonolides are not oxidized at C11 such as taccalonolides E and N. In the case of AE, a rare germinal diol is formed at C7. In addition to the ketone group at C6 and C7, taccalonolides H and M have a second ketone group at C7 and C15, respectively. Another major contributor to the diversification of taccalonolides is the breakage of the enol-γ-lactone ring and the formation of δ-lactone between C15 and C24 or C22 and C24, which is seen in taccalonolides C, X, Q, and Y. Additionally, taccalonolide H_2_ and Taccalonolide AD have an uncommon unsaturated ring B with a double bond at C5,6 and C7,8, respectively (Figure 2). Taccalonolides I, J, and K are distinguished from other taccalonolides by the shift of the ketone group on ring B that is typically located at C6 to C7 (Table 2). 

All taccalonolides isolated, their source plants, and the activities were summarized in Table 1. Taccalonolides are only found from those plants in *Tacca* species. Thus far, a total of 44 taccalonolides have been identified from five species of plants, *T. plantaginea, T. chantrieri, T. paxiana*, *T. integrifolia*, and *T. subflaellata.* The effects of bundling of interphase microtubules, the formation of multiple aberrant mitotic spindles, and antiproliferative actions were observed for most of the taccalonolides. 

Taccalonolides A and E were the first taccalonolides identified with microtubule-stabilizing activity in the year 2003 and a series of naturally occurring taccalonolides were isolated in the succeeding years, in search of more potent structures (Table 1). Taccalonolides A and E had played an important role in early studies of the interactions of taccalonolides and microtubules due to their abundant occurrence until the discovery of the highly potent taccalonolide AF in the year 2011 [16]. The structure of taccalonolides AF was proposed as the C22–C23 epoxide of taccalonolides A, based on the spectra of a very minor and impure material. Due to the extraordinary potency of the fraction, taccalonolides AF was synthesized through epoxidation of taccalonolide A, which proved the proposed structure of taccalonolides AF and its bioactivity. Taccalonolide AF marked a milestone in understanding the structure-activity relationships (SARs) of taccalonolides and the mechanism of action of this class of compounds. The absolute configuration of the C22–C23 epoxide of taccalonolides AF and AJ was determined to be *R*,*R*, by single-crystal X-ray diffraction analysis [17,18].

**Figure 3:**
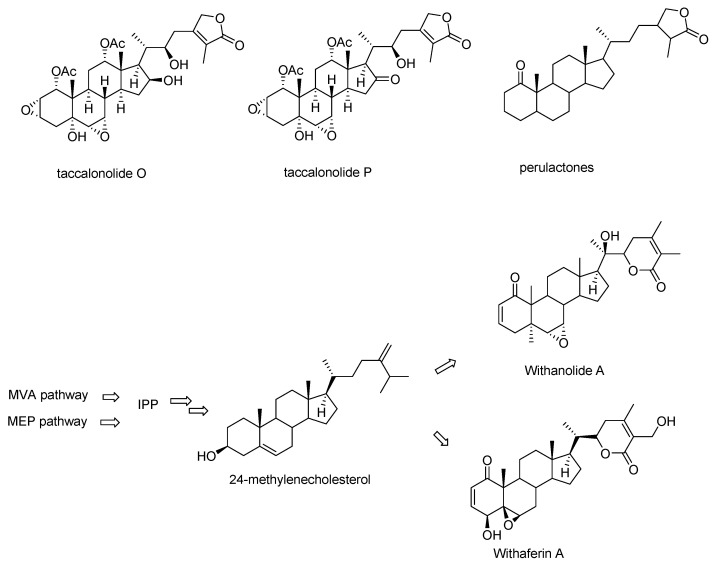
The Structures of taccalonolides O and P, the basic structure of perulactones, and an overview of withanolide biosynthesis.

Taccalonolides O and P, which lack the carbon-carbon bond between C16 and C24 resulting in the absence of the characteristic E ring were categorized into taccalonolides in the original publications but listed as perulactones in a late review (Figure 3) [15,19,20,21]. Taccalonolides O and P are structurally closer to perulactones, so they are not included in the list of taccalonolides in this review. Taccalonolides and perulactones are often included as two subclasses in reviews or books on withanolides while sometimes taccalonolides are also listed as an independent class from withanolides [20,21,22]. Withanolides are a large group of steroidal lactones with a wide range of pharmacologic properties found in the Indian medicinal plant *Withania somnifera*. They are also found in plants of many members of the family *Solanaceae* and in the genus *Tacca* [19,20,23]. The common feature of withanolides is an intact or rearranged ergostane with a highly structurally diverse side chain (Figure 3). The pathways for withanolide biosynthesis have been well-studied, in particular for the arrangement of the side chain in recent years [24,25,26]. Though no genes or enzymes specifically responsible for taccalonolides biosynthesis have been characterized to date, the fact that taccalonolides, perulactones, and withanolides are co-isolated from various plants suggesting that they share the same upstream biosynthetic pathway [21,23,27].

## 3. Semisynthetic Taccalonolides and Structure-Activity Relationships (SARs) 

**Figure 4:**
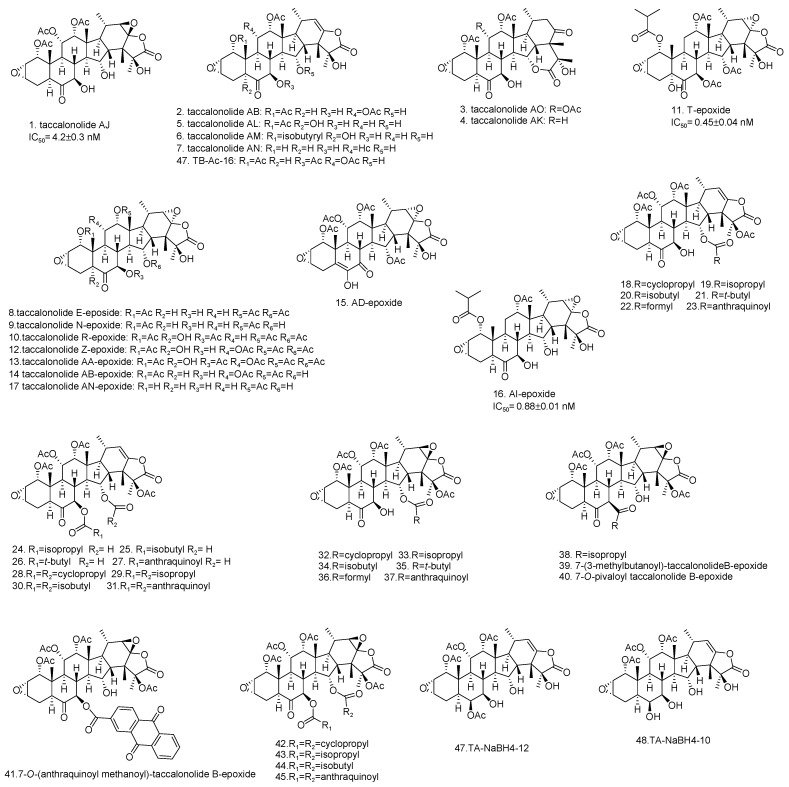
The structures of semi-synthetic taccalonolides (1–48).

**Figure 5:**
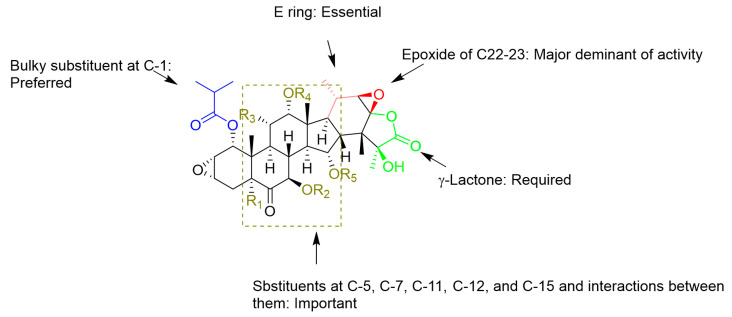
Structure-activity relationships (SARs) of reported taccalonolides.

Among the taccalonolides obtained from plants, taccalonolides AF, AI, and AA exhibited the most potent in vitro antiproliferative action on a HeLa cervical cancer cell line with IC_50_ values of 23, 47, and 32 nM, respectively [16,30,42]. The structure of taccalonolide AF differs from taccalonolide A just by converting the C22–23 double bond in taccalonolides A to an epoxy group in taccalonolides AF. Inspired by the potent activity of AF, taccalonolide AJ was semi-synthesized by epoxidation of another abundant component, taccalonolide B (Table 3). The activity of AJ is 743-fold higher than that of taccalonolide B (Table 4) [16]. To further explore the role of the epoxyl for microtubule stabilization activity, a click epoxidation reaction was developed using dimethyldioxirane as an epoxidation agent. This reaction allowed the semi-synthesis of epoxide derivatives (<1 mg) of rare natural taccalonolides, which confirmed the finding that epoxidation of the C22–23 double bond is an effective way to increase the potency of this class of molecules (Table 3 and Figure 4) [42]. In contrast, the rearrangement of γ-lactone to δ-lactone in the case of taccalonolides AO, AK, and AT-AY and the presence of an α-hydroperoxyl group at C20 of taccalonolide AC led to the lack of cytotoxicity and microtubule-stabilizing activities [23,31]. It can be concluded that the E-ring region of taccalonolides is critical for microtubule-stabilizing activities (Figure 5).

Taccalonolides AI and N differ only at the C1 position, with an isovaleryl group in taccalonolide AI and an acetyl group in taccalonolide N, but they show significant differences in antiproliferative efficacy [39]. Similarly, taccalonolides R and AL exhibit much lower antiproliferative activities than their isovaleric analogs, taccalonolides T and AM. Interestingly, hydrolysis of the C-1 acetoxy group in taccalonolide N generates taccalonolide AN, which slightly increases the potency. These observations illustrate the importance of the C-1 substituent and especially its size. Consistent with the conclusions above, the combination of esterification with an isovaleryl group at C-1 and epoxidation at C22–C23 provided the most potent taccalonolides to date, AI-epoxide and T-epoxide, with IC_50_ values of 0.88 nM and 0.45 nM, respectively. To further examine the effect of bulky substituents on the potency of biological activity, 14 new taccalonolides with substitutions at C-7, C-15, and/or C-25 along with their C22-23-epoxy derivatives were semi-synthesized [39]. The evaluation of the in vitro bioactivities indicated that a longer, less branched-chain at either C-7 (**26**) or C-15 (**21**) leads to modestly increased potency. 

Unlike taccalonolides AF and AI, taccalonolide AA does not have an isovalery or an epoxy group. Its superior potency cannot be simply explained by a single change of the structure, which indicates a complex combinatory contribution of multiple functional groups [30]. For example, it appears that the presence of the hydroxyl group at C-5 is associated with a higher antiproliferative activity by comparing the activity of taccalonolide A with that of its corresponding compound, taccalonolide Z, which contains a C-5 hydroxyl group. Conversely, the potencies of taccalonolides AM, AL, and AB with the C-5 hydroxyl moiety decrease by a range from 42.5- to 1.1-fold in comparison with taccalonolides AI, N, and B. The only structural difference between the two groups of taccalonolides that show an opposite tendency regarding the effect of the C-5 hydroxyl group is a hydroxyl group or an acetoxy at C-15, suggesting combined interactions between substituents at the sites of C-5 and C-15 with the tubulin. A similar influence between the substituents at C-7, C-11, and C-15 are observed as well. The complex relationships among multiple sites on the taccalonolide backbone have been discussed thoroughly [30]. Further efforts to unravel and understand the relationships between different constituents on the backbone regarding their effects on the biological activity may be an effective avenue to discover more potent taccalonolides. In addition, among the other changes in the B ring such as keto-enol tautomerization at C6-C7 (taccalonolide B vs taccalonolide I), dehydrogenation (taccalonolide A vs taccalonolides AD and H_2_) or the formation of a geminal diol group (taccalonolides AE), the 6,7-double bond (taccalonolides H_2_) moderately increase the activity [16,30].

**Figure 6:**
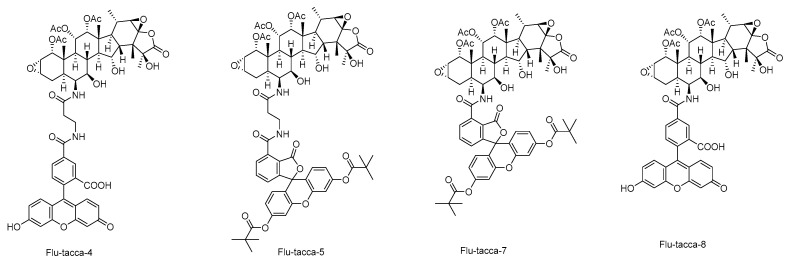
The selective structures of taccalonolide probes.

These SARs are observed as well by computational analysis such as i) taccalonolides covalently bind to β-tubulin D226 via the C22–C23 epoxide group, and ii) the bulky group at C-1 is involved in interactions with β-tubulin residues through hydrogen bonds [17,18,45]. According to established SARs, a group of taccalonolides with a fluorescein group at C-6 such as Flu-tacca-4, Flu-tacca-5, Flu-tacca-7, and Flu-tacca-8 were designed and generated to facilitate the identification of binding site interactions, among which Flu-tacca-7 showed comparable potencies in the proliferation of HeLa and SK-OV-3 cells (Figure 6) [18,46].

## 4. Pharmacological Effects and Mechanism of Action of Taccalonolides

### 4.1. Cellular Actions on Interphase Microtubules, Mitosis, and Cell Cycle

When cancer cells (A-10 and HeLa cells and human lung carcinoma A549 cells) are treated with taccalonolides, the density of cellular microtubules increase and the microtubule network rearranges into short bundles or tufts of thick microtubules in a concentration-dependent way [13,28]. Taccalonolide A begins to cause interphase microtubule bundles in HeLa cells at 250 nM, which is much less than its IC_50_ value of 5380 nM for the same cell line. The number and thickness of the bundles noticeably increase with 500 nM, 1 μM, and 2.5 μM taccalonolide A [28]. The bundled microtubules are denser around the nucleus, but also can be found around the cell periphery. Moreover, similar to paclitaxel and laulimalide, taccalonolides cause the breakdown of the nucleus into multiple micronuclei in mitotic cells [13].

A few subtle differences are observed between the effects of taccalonolides and paclitaxel on interphase microtubules. Taccalonolides-induced microtubule bundles are generally short and nucleate independently of the centrosomes, while Paclitaxel-initialed microtubules consistently appear to be long and fill more of the cytoplasm [13]. Taccalonolides require a much lower relative concentration to induce interphase microtubule bundling than paclitaxel and laulimalide [28]. For example, 250 nM taccalonolide A is required to initiate interphase microtubule bundling, which was less than its IC_50_ value of 5940 nM. In comparison, paclitaxel requires a concentration of 50 nM to initiate microtubule bundling in the same cell line, which is 31 fold of its IC_50_ value of 1.6 nM [28]. 

Microtubule dynamics is faster during mitosis compared to the interphase, thus mitotic cells are very sensitive to tubulin-interacting drugs. Taccalonolides E and A concentration-dependently cause the appearance of abnormal multipolar mitotic spindles in a concentration dependent manner in A-10 and HeLa cells. The abnormal spindles appear ineffective in aligning the DNA in metaphase [13]. Cells treated with up to 500 nM taccalonolide E formed normal bipolar spindle poles. At a concentration of 1 µM taccalonolide E, the majority of the mitotic cells have three or more spindle poles and 70% of them have five or more spindle poles at 5 µM concentration [13]. The taccalonolide-initiated formation of abnormal mitotic spindles causes cells to accumulate in the G2-M phase of the cell cycle with 4N DNA content, which is widely accepted to be the mechanism of inhibiting the proliferation of cancer cells by microtubule-targeting agents [13,30]. The appearance of the sub-G1 peak presumably of cells undergoing apoptosis is observed in taccalonolide-treated cells [13,47]. These findings are consistent with the molecular events initiated by taccalonolides including the phosphorylation of Bcl-2 and activation of Caspase 3 and MAPK-signaling pathways [13].

Effects of the potent AF and AJ on interphase and mitotic microtubules are similar to those previously observed with less potent taccalonolides. Similarly, microtubule bundling is observed at the IC_50_ concentration of taccalonolides AF and AJ [40]. Taccalonolide AJ initiated abnormal mitotic spindles containing five to nine small, compact spindle asters that are located throughout the cytoplasm, distinctive in both morphology and the number of asters as compared to cells treated with laulimalide or paclitaxel [48]. The majority of the paclitaxel-treated cells contain a single large, diffuse aster that is often accompanied by two smaller, more punctate asters [49]. Microtubule stabilizer-treated cells contain only two γ-tubulin foci and two pairs of centrin signals, associated with only two of the many spindle asters. In contrast, multiple pericentrin foci are observed in taccalonolide AJ-treated cells but not in paclitaxel or laulimalide treated cells. In addition, taccalonolides have a higher propensity for centrosomal separation defects than other microtubule stabilizers [48]. Alteration of the expression, activation, and localization of central mitotic kinases including Plk1, Aurora A, and TPX2 by chemically diverse microtubule stabilizers may be a mechanism by which microtubule stabilizers initiated distinct aberrant mitotic spindles and centrosomal separation defects [48]. The distinct mitotic microtubule morphology might be partially explained as well by the distinct ability of the taccalonolides to inhibit microtubule shortening and catastrophe that caused the inability of multiple microtubule asters to coalesce [49].

### 4.2. In Vitro Antiproliferative Effects and In Vivo Antitumor Efficacy

All isolated taccalonolides exhibit cytotoxicity toward cancer cells (HeLa cells) with a range of potencies from low nanomolar to micromolar levels, except for taccalonolides D and AC [16,30]. An advantage of taccalonolides over the taxanes is their ability to circumvent multiple drug resistance mechanisms. An early study on the evaluation of antiproliferative efficacy of taccalonolides shows that taccalonolides E and A inhibit the proliferation of drug-sensitive cancer cell lines SK-OV-3 and MDA-MB-435, albeit less potent than paclitaxel. However, excitingly, the multidrug-resistant cell lines NCI/ADR, the taxol-resistant cell lines, PTX 10, PTX 22, and the epothilone-resistant cell line, 1A9/A8, are all less resistant to the taccalonolides E and A [13]. In a later study, the susceptibilities to cellular resistance mechanisms including overexpression of P-glycoprotein, MRP7, and the βIII isotype of tubulin are evaluated. Taccalonolides A, E, B, and N are significantly better than paclitaxel at circumventing Pgp-mediated drug resistance both in vitro and in vivo. All four tested taccalonolides also overcome resistance due to the expression of MRP7 and β-tubulin isotypes [33]. 

A recent study suggests that inhibition of the activation of the sonic hedgehog (Shh) pathway also contributes to the antiproliferative effects of taccalonolide A against Hepatocellular carcinoma (HCC) cells [32]. Taccalonolide A represses cell viability and accelerates apoptosis of HepG2 and Huh7 cells. It is observed that mRNA and protein expression levels of Shh, SMO and GlI1 were decreased in taccalonolide A treated HCC cells (Figure 1b).

It is found that taccalonolides A and E are substantially more potent in vivo than would be expected from their in vitro potency [33]. Additionally, the in vivo evaluation suggests that taccalonolides have narrow therapeutical windows. For instance, a dose of 56 mg/kg taccalonolide A providing the longest tumor growth delay and the highest gross log cell kill is above the maximum tolerated dose. The same effects are observed with more potent taccalonolide AF [30,40]. Taccalonolide AF is found to have potent antitumor effects with inhibition of tumor growth almost identical to that of 10 mg/kg paclitaxel when a 2.0 mg/kg dose is administered. A slightly higher dose of 2.5 mg/kg with a cumulative dose of 5.0 mg/kg causes significant weight loss and toxicity. The lack of antitumor efficacy at doses lower than the LD_80_ leads taccalonolide AJ absent of any therapeutic window in the MDA-MB-231 breast cancer xenograft model [40]. However, AJ has excellent and persistent antitumor efficacy when it is intratumorally injected into the tumor. Its short half-life (*t*_1/2_) of 8.1 min limits its delivery to the tumor and precluded it from achieving antitumor efficacy at systemically tolerable doses [50]. A cyclodextrin inclusion complex of AJ has been developed to improve the therapeutic window and reduced toxicity [51]. It is also observed that the mitotic arrest and the antiproliferative effects of taccalonolides are more persistent and less reversible than the other microtubule disrupting agents evaluated, paclitaxel and laulimalide [28]. Similarly, two potent semi-synthetic taccalonolides, bearing isovalerate modifications at C-7 or C-15, demonstrate highly persistent in vivo antitumor activity in a drug-resistant xenograft model [39].

### 4.3. Tubulin Polymerization and Microtubule Stabilization Caused by Taccalonolides

Taccalonolide E is found to rapidly cause the in vivo polymerization of tubulin in an early study [13]. However, Taccalonolides E and A are unable to polymerize tubulin in biochemical extracts even in the presence of a full complement of cytosolic proteins or in cellular lysates [28,47]. Direct interaction of taccalonolides with tubulins/microtubules were not identified until the discovery of taccalonolides AF and AJ, which promoted polymerization of purified tubulin to the same extent as paclitaxel at equimolar concentrations [16]. A lag period of 4 min is observed with 0.1 µM paclitaxel or 0.25 µM laulimalide, while polymerization occurres immediately after the addition of 5 µM of either drug. Unlike paclitaxel and laulimalide, a lag of at least the 5 min required for polymerization to occur is observed over the entire range of taccalonolides AJ concentrations, implying a novel binding site [16]. Taccalonolide AJ enhances the rate and extent of tubulin polymerization in a manner that is markedly different from either paclitaxel or laulimalide. A concentration of 10 μM taccalonolide AJ results in a 4.7-fold increase in polymerization rate over the vehicle and a doubling in total polymer formed, which is almost identical to the effect of 10 μM paclitaxel or laulimalide. Higher concentrations of 20 or 30 μM AJ cause further increases (30–66%), in both the rate of polymerization and the total polymer formed while those values do not increase significantly with higher concentrations of paclitaxel or laulimalide [40].

Taccalonolides impart robust cold and mechanical stability to microtubules compared to paclitaxel and laulimalide. Taccalonolide AJ or AF-induced microtubules are insensitive to dramatic or mild cold-induced depolymerization and once the reaction is rewarmed, a further increase in turbidity is observed [40].

### 4.4. Define the Covalent Binding 

The differences between the effects of taccalonolides and other microtubule-stabilizing agents on interphase microtubules and mitotic spindles, the efficacy against multidrug-resistant tumors, and the high degree of cellular persistence after drug washout suggest that taccalonolides have a unique binding site and a distinct mechanism of action [28,30,47]. The ability of AF to cause synergistic antiproliferative effects in combination with paclitaxel or laulimalide as determined by isobologram analysis and calculation of combination indices indicate the existence of non-overlapping binding sites. After incubation with equal moles of purified tubulin, only 7% of AJ remain in the supernatant and no drug is extracted from the microtubule pellet even with stringent extractions, indicating the possibility of a covalent interaction of AJ with microtubules. In another experiment, mass spectrometry is utilized to characterize the interaction between the taccalonolides with tubulin/microtubules. In contrast to the detection of the mass of β213-230 peptide in the control, a mass corresponding to β213-230 plus AJ is detected, confirming that AJ covalently binds to these peptides on β-tubulin [40]. Hydrogen/deuterium exchange mass spectrometry is employed to further probe the allosteric effects elicited by this covalent interaction and demonstrates that AJ induces a markedly higher stabilization of the lateral inter-protofilament contacts centering on α-tubulin in a manner that does not appear to involve the M-loop [40]. However, later computational analysis shows AJ binding is compatible with maintaining the M-loop in a helical conformation and noncovalent interactions between the taccalonolide fluorescent probe, Flu-tacca-5 and Flu-tacca-8, and β-tubulin residues involve M-loop [17,18].

In an initial experiment to examine the exact amino acid residues involved in AF or AJ covalent binding, a 2.05Å crystal structure of the AJ–tubulin complex (PDB ID: 5EZY) is determined [42]. The structure reveals that AJ forms hydrogen bonds with β-tubulin D226, H229, T276, and R278, and a weak hydrogen bond with K19. More importantly, AJ covalently binds to β-tubulin D226 via the C22–C23 epoxide group, which is consistent with the above mass spectrometry data and explains the critical role of the C22–C23 epoxide group showed in the SARs analysis. Besides the covalent binding of the C22–C23 epoxide group, analysis of the docking model for β-tubulin complexed with AI epoxide discloses that several β-tubulin residues (L217, L230, L275, and F272) interact with the bulky isovaleryloxy group at C-1, which may be the reason of the preferred bulky group at C-1 for the potency [45]. 

**Figure 7:**
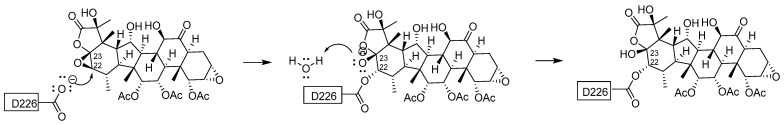
Proposed mechanism for the addition of Asp226 to taccalonolide AJ.

An SN2 reaction mechanism of the 1,2-addition of Asp226 to AJ is supported by means of QM/MM sMD simulations and by covalent docking using CovDock (Figure 7) [17,18]. The nucleophilic carboxylate of Asp226 attacks the C22 of the C22-C23 epoxide in AJ and the opened epoxide ring.

Based on binding energy analysis, residues of β-tubulin His229, Leu217, Leu219, Leu230, Leu371, Pro360, Phe272, and Leu275 are identified as a determinant of the orientation of AJ in the [TAJ:(α1-β1-α2)]_2_ complex. His229, Asp226, Arg278, Lys19, and Arg369 are in favorable contact with AJ. The simulation reveals that the hydrogen bonds are formed between the C2–C3 epoxide and the side chains of Lys19 and Glu22 in helix H1 as well as Asp26 and Glu27 [17]. The C6 carbonyl and the oxygen of the C1 acetyl moiety also engage in a long-lived water-mediated hydrogen bond to Asp26 and Pro274, respectively [17]. 

**Figure 8:**
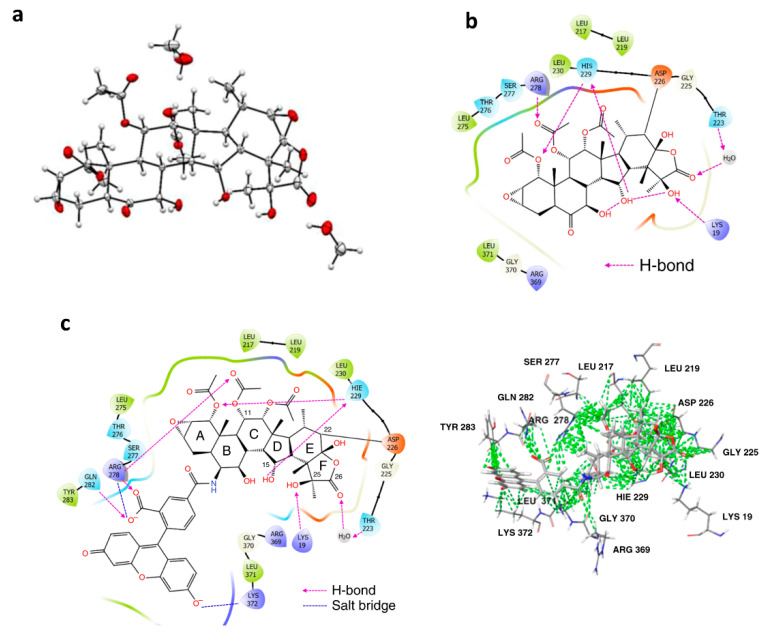
Taccalonolide microtubule stabilizers covalently bind to β-tubulin (cited from reference [18] without changes, http://creativecommons.org/licenses/by/4.0/ accessed on 21 January 2021). (**a**). ORTEP drawing of the single-crystal X-ray structure of AJ; (**b**). Key tubulin residues that mediate the binding affinity of AJ in a docking model structure generated by CovDock; (**c**). Key tubulin residues that mediate the binding affinity of the fluorogenic taccalonolide probe in the docking model structure.

Analysis of the 5EZY crystal structure and covalent docking models of taccalonolide AJ and Flu-tacca-8 suggests that, besides D226, six additional residues of β-tubulin (i.e., H229, R278, K19, Q282, T223, and K372) are likely to play important roles in interaction with taccalonolide via H-bond and/or salt bridges and hydrophobic interactions. Nine additional β- tubulin residues (i.e., L217, R369, L219, L230, L371, Y283, G225, G370, and S277) only have hydrophobic interactions (Figure 8) [18]. Another fluorogenic taccalonolide probe Flu-tacca-7 is successfully generated and used with mutagenesis by immunoblotting to determine the target specificity of the taccalonolides. Thus, the impact of β-tubulin residues D226, K19, H229, R278, L217, L219, and T223 on taccalonolide binding are confirmed along with their quantified relative contributions [18].

## 5. Conclusions

Taccalonolides are the first class of covalently bound microtubule stabilizers displaying potent anticancer properties and, undoubtedly, are a group of attractive therapeutic candidates for cancer treatment [15]. Taccalonolides have shown excellent efficacy in drug-resistant models both in vitro and in vivo. Long-lasting therapeutic responses and high cellular persistence of taccalonolides are observed in murine xenograft models and clonogenic assay. One of the major challenges to further develop taccalonolides for clinical use is that taccalonolides has a narrow therapeutic window when administered systemically. The potent fluorescein-labeled taccalonolides synthesized in this study suggest that targeted drug delivery, such as by attaching an antibody to taccalonolides, might be an efficacious strategy to reduce the optimal drug dose required for a significant antitumor effect and manage systemic toxicities [18,46]. In the last several years, understanding of SARs and the mechanism of action of taccalonolides has advanced significantly, which will facilitate the discovery and designing of new chemical entities with potent and selective anti-cancer activities. In summary, as a new generation of microtubule stabilizers, taccalonolides have shown promising potential for the treatment of cancers with exciting advantages over current anticancer agents.

## Figures and Tables

**Table 1:** Natural taccalonolides listed chronologically based on their first appearance in publications.

No.	Compound Name	Species	Biological Activities (Year)	Ref.
1	Taccalonolide A	*T. plantaginea,* *T. chantrieri,* *T. paxiana*	Bundling of interphase microtubules in HeLa cells at 250 nM. (2011) 5 µM taccalonolide A induced bundles, multipolar spindles, and multiple micronuclei on Human lung carcinoma A549 cells. (2005) Initiating Bcl-2 phosphorylation, MAPK activation, and apoptosis. (2003) In vivo antitumor effects on the syngeneic murine mammary carcinoma 16/C model. (2011) Inhibiting cell proliferation of two drug-sensitive cell lines SK-OV-3 and SK-OV-3/MDR-1-6/6 with IC_50_s of 0.6 µM and 2.5 µM. (2008) Inhibiting cell proliferation of taxol-resistant cell lines, PTX 10 and PTX 22 with IC_50_s of 7.05 µM and 6.40 µM, and the epothilone-resistant cell line, 1A9/A8 with IC_50_ of 8.89 µM. (2003) Inhibiting proliferation of HepG2 and Huh7 cells with IC_50_ values for HepG2 and Huh7 cells 11.9 µM and 16.8 µM, respectively. (2020) In vivo against a doxorubicin- and paclitaxel-resistant Pgp-expressing tumor in syngeneic mammary 17/ADR model. (2008)	[12,13,16,28,29,30,31,32,33]
2	Taccalonolide B	*T. plantaginea,* *T. paxiana*	Antiproliferative effects in HeLa cells. (2011) Inhibiting cell proliferation of two drug-sensitive cell lines SK-OV-3 and SK-OV-3/MDR-1-6/6 with IC_50_s of 0.2 µM and 2.5 µM. (2008) Mitotic arrest and bundling of interphase microtubules in HeLa cells at 0.8 μM. (2011)	[12,16,29,30,31,33]
3	Taccalonolide C	*T. plantaginea*	N/A ^1^	[34]
4	Taccalonolide D	*T. plantaginea*	N/A	[34]
5	Taccalonolide E	*T. plantaginea* *T. chantrieri,* *T. paxiana,*	Mitotic accumulating in the G2-M phase of the cell cycle. (2003, 2005) Forming of multiple aberrant mitotic spindles and initiating micronucleation in interphase A-10 cells at 1µM. (2003) Bundling of interphase microtubules. Antiproliferative effects in HeLa cells. (2003) Inhibiting cell proliferation of two drug-sensitive cell lines SK-OV-3 and SK-OV-3/MDR-1-6/6 with IC_50_s of 0.7 µM and 3.6 µM. Inhibiting cell proliferation of taxol-resistant cell lines, PTX 10 and PTX 22 with IC_50_s of 1.64 µM and 4.01 µM, and the epothilone-resistant cell line, 1A9/A8 with IC_50_ of 1.42 µM. (2003, 2008) In vivo antitumor effects on the syngeneic murine mammary carcinoma 16/C model. (2011) In vivo against a doxorubicin- and paclitaxel- resistant Pgp-expressing tumor in syngeneic mammary 17/ADR model. (2008)	[13,29,30,31,33,35]
6	Taccalonolide F	*T. plantaginea*	N/A	[35]
7–8	Taccalonolides L and M	*T. plantaginea*	N/A	[36]
9–11	Taccalonolides G, H, and J	*T. plantaginea*	N/A	[37]
12	Taccalonolide I	*T. plantaginea*	Antiproliferative effects in HeLa cells (2013)	[31,37]
13	Taccalonolide K	*T. plantaginea,* *T. paxiana*	N/A	[29,37]
14	Taccalonolide N	*T. paxiana*	Antiproliferative effects in HeLa cells. (2013) Inhibiting cell proliferation of two drug-sensitive cell lines SK-OV-3 and SK-OV-3/MDR-1-6/6 with IC_50_s of 0.2 µM and 1.2 µM. Mitotic arrest and bundling of microtubules in HeLa cells at 1.3 μM. (2011) In vivo antitumor effects on the syngeneic murine mammary carcinoma 16/C model. (2011)	[29,30,31,33]
15	Taccalonolide Q	*T. subflaellata*	N/A	[21]
16	Taccalonolide R	*T. paxiana* *T. chantrieri*	Mitotic arrest and bundling of microtubules in HeLa cells at 57 μM. (2011)	[29,30]
17	Taccalonolide S	*T. paxiana*	N/A	[29]
18	Taccalonolide T	*T. paxiana* *T. chantrieri*	Antiproliferative effects in HeLa cells. Mitotic arrest and bundling of microtubules in HeLa cells at 3.5 μM. (2011)	[29,30]
19–20	Taccalonolide U and V	*T. paxiana*	N/A	[29]
21–23	Taccalonolides W, X, Y	*T. plantaginea*	N/A	[38]
24	Taccalonolide Z	*T. integrifolia*	Antiproliferative effects in HeLa cells using the SRB assay. Mitotic arrest and bundling of microtubules in HeLa cells at 0.6 μM. (2011)	[30]
25	Taccalonolides AA	*T. chantrieri*	Antiproliferative effects in HeLa cells. (2011) Mitotic arrest and bundling of interphase microtubules in HeLa cells at 0.32 μM. (2011)	[30]
26	Taccalonolides AC	*T. plantaginea*	Lack of potency (IC_50_>50,000 nM against HeLa cells). (2013)	[16,31]
27–28	Taccalonolides AD-AE	*T. plantaginea*	Increasing in cellular microtubule density and microtubule bundling in HeLa cells at 17 and 25 μM. Antiproliferative actions in HeLa cells with IC_50_ of 3.48 and 5.01 μM. (2011)	[16]
29	Taccalonolid AF	*T. plantaginea*	Increasing the density of interphase microtubules HeLa cells. (2011) Causing HeLa cells to arrest in the G2/M phase of the cell cycle with multiple aberrant mitotic spindles at 100 nM. (2011) Stimulating the polymerization of purified tubulin. (2011) High antiproliferative potency in HeLa cells. (2011) In vivo antitumor effect in the MDA-MB-231 breast cancer xenograft model. (2013)	[16,39,40]
30	Taccalonolides H_2_	*T. plantaginea*	Increasing the density of interphase microtubules HeLa cells. Causing HeLa cells to arrest in the G2/M phase of the cell cycle with multiple aberrant mitotic spindles. Antiproliferative potency in HeLa cells using the SRB assay. (2011)	[16]
31	Taccasuboside A	*T. subflabellata*	Lack of potency (2011)	[41]
32	Taccalonolid AI	*T. chantrieri*	Potent antiproliferative effect in HeLa cells. (2014)	[42]
33–38	Taccalonolides AT-AY	*T. chantrieri*	Devoid of cytotoxicity. (2015)	[23]
39	Taccalonolide A 12-propanoate	*T. leontopetaloides*	Antitrypanosomal activity against Trypanosoma brucei brucei with the EC_50_ value of 3.13 + 0.089 μg/mL. (2016)	[43]
40–41	Taccalonolides AG, AH	*T. chantrieri*	Antiproliferative in HeLa cells. (2019)	[44]
42–44	Taccalonolides AP, AQ, and AR	*T. chantrieri*		

^1^ N/A: Not available.

**Table 2:** Selective natural taccalonolides with rare substructures or substituents.

Substituent	Compound Name
11-H	taccalonolides E, G, N, U, AI, AG, AH, AP
6-OH or 6-OAc	Taccalonolide I, J, K, M, AD
6=O and 7=O	Taccalonolide H
7=O and 15=O	Taccalonolide M
20-OOH	Taccalonolide AC
δ-lactone between C15 and C24	Taccalonolides Q, Y
δ-lactone be-tweenC22 and C24	Taccalonolides C, X, AY, AW, AT, AU, AV, AX,
unsaturated ring B	Taccalonolides H_2_ and AD
unsaturated ring E	Taccalonolides M, F, AQ, AR and
No δ-lactone or γ-lactone on E ring	Taccasubosides A

**Table 3:** Semi-synthetic taccalonolides based upon naturally occurring molecules.

No.	Compound Name	Biological Activities (Year)	Ref.
1	Taccalonolide AJ	Stimulating the polymerization of purified tubulin. (2011) Increasing the density of interphase microtubules in HeLa cells at 30 nM. (2011) Causing HeLa cells to arrest in the G2/M phase of the cell cycle with multiple aberrant mitotic spindles. (2011) High antiproliferative potency in HeLa cells. (2011) In vivo antitumor effect in the MDA-MB-231 breast cancer xenograft model with the absence of therapeutic window. Taccalonolide AJ showed excellent antitumor efficacy only when directly administered to the tumor. (2013)	[16,39,40]
2	Taccalonolide AB	Mitotic arrest and bundling of interphase microtubules in HeLa cells at 13.5 μM. (2011) Antiproliferative action in HeLa cells. (2014)	[30,42]
3–4	Taccalonolides AO, AK	Lack of potency (IC_50_>50 μM against HeLa cells). No microtubule stabilizing effects at 50 μM. (2013)	[31]
5–7	Taccalonolides AL, AM, AN	Antiproliferative actions in HeLa cells. Increase in the cellular density of interphase microtubules in HeLa cells at 10-50 μM. (2013)	[31]
8–17	Taccalonolide E-epoxide, Taccalonolide N-epoxide, Taccalonolide R-epoxide, Taccalonolide T-epoxide, Taccalonolide Z-epoxide, Taccalonolide AA-epoxide, Taccalonolide AB-epoxide, Taccalonolide AD-epoxide, Taccalonolide AI-epoxide, Taccalonolide AN-epoxide,	Causing interphase microtubule bundling in HeLa cells. Antiproliferative activities in HeLa cells. Taccalonolide T-epoxide and Taccalonolide AI-epoxide enhanced polymerization of purified tubulin in turbidimetric assays. Taccalonolide T-epoxide and Taccalonolide AI-epoxide possess antitumor efficacy in the MDA-MB-231 triple negative breast cancer xenograft mice model. (2014)	[42]
18–45	7-(3-methylbutanoyl)-taccalonolide B-epoxide and other C-7, C-15, and C-7, C-25 acyloxy taccalonolide B and their corresponding C-22, C-23 epoxides	Antiproliferative effects against the HeLa cell line. Eliciting the tubulin polymerization in HeLa cells. In vivo antitumor activities in female athymic nude mice with transplanted NCI/ADR-RES tumor fragments. (2018)	[39]
46	TB-AC-16	Antiproliferative effects in HeLa cells. (2019)	[45]
47	TA-NaBH4-12		
48	TA-NaBH4-10		
49–56	Flu-tacca-1 ~Flu-tacca-8	Causing a decrease in the proliferation of HeLa or SK-OV-3 cells. Polymerizing purified tubulin. Binding to endogenous β-tubulin of HCC1937 cells evaluated by immunoblotting. (2020)	[18]

**Table 4:** Antiproliferative potencies of taccalonolides [15,18,39,42].

Compound	IC_50_ (nM) ^1^	Compound	IC_50_ (nM)
Taccalonolide A	5380 ± 230	Taccalonolide AE	5010 ± 210
Taccalonolide B	3120 ± 180	Taccalonolide AF	23 ± 3
Taccalonolide I	49,200 ± 2800	Taccalonolide AJ	4.2 ± 0.3
Taccalonolide T	335 ± 24	Taccalonolide H_2_	730 ± 20
Taccalonolide R	13,144 ± 1390	Taccalonolide AL	34,400 ± 7500
Taccalonolide E	39,500 ± 4700	Taccalonolide AO	>50,000
Taccalonolide N	8500 ± 400	Taccalonolide AK	>50,000
Taccalonolide Z	120 ± 7.5	Taccalonolide AM	2000 ± 100
Taccalonolide AA	32.3 ± 1.9	Taccalonolide AN	1500 ± 100
Taccalonolide AB	2767 ± 107	Taccalonolide AI	47 ± 3
Taccalonolide AC	>50,000	Taccalonolide AI-epoxide	0.88 ± 0.01
Taccalonolide AD	3480 ± 230	Taccalonolide T-epoxide	0.45 ± 0.04
Taccalonolide Z-epoxide	17.2 ± 0.3	Taccalonolide AN-epoxide	685 ± 19
7- *O*- pivaloyl taccalonolide B-epoxide	9 ± 1	7-*O*-(anthraquinoyl methanoyl)-taccalonolide B-epoxide	22 ± 3
7-*O*-(3-methylbutanoyl)-taccalonolide B-epoxide	2.4 ± 0.7	Flu-tacca-7	31 ± 2

^1^ The concentrations of taccalonolides that caused 50% inhibition of cellular proliferation (IC_50_) were measured in HeLa cells using the SRB assay (*n* = 3).
